# Persistent depression in pregnant refugee and migrant women living along the Thai-Myanmar Border: a secondary qualitative analysis.

**DOI:** 10.12688/wellcomeopenres.17744.2

**Published:** 2024-03-28

**Authors:** Tabitha Ashley-Norman, Gracia Fellmeth, Tobias Brummaier, Suphak Nosten, May May Oo, Yuwapha Phichitpadungtham, Kerry Wai, Napat Khirikoekkong, Emma Plugge, Rose McGready

**Affiliations:** 1 School of Medicine, University of Leeds, Leeds, LS2 9JT, UK; 2 Shoklo Malaria Research Unit, Oxford Tropical Medical Research Unit, Faculty of Tropical Medicine, Mahidol University, Mae Sot, 63100, Thailand; 3 Nuffield Department of Population Health, University of Oxford, Oxford, OX3 7LF, UK; 4 Swiss Tropical and Public Health Institute, Allschwil, CH-4123, Switzerland; 5 University of Basel, Basel, CH-4001, Switzerland; 6 Faculty of Medicine, University of Southampton, Southampton, SO17 1BJ, UK; 7 Centre for Tropical Medicine and Global Health, Nuffield Department of Medicine, University of Oxford, Oxford, OX3 7LG, UK

**Keywords:** maternal, antepartum depression, refugee, migrant, mental health, marginalised, SCID, displaced populations

## Abstract

**Background:**

Antepartum depression affects around 15% of pregnant women worldwide, and may negatively impact their infants’ physical, cognitive and social development, and confer a greater risk of emotional dysregulation in their children. Risk factors for antepartum depression disproportionately affect women from resource-sparse settings. In particular, pregnant refugee and migrant women face many barriers to diagnosis and care of mental health conditions, yet this group is under-represented in the literature. This study explores what refugee and migrant women living along the Thai-Myanmar border perceive as being contributory and protective factors to their antepartum depression, through secondary qualitative analysis of responses to clinical interviews for depression.

**Methods:**

Previous research investigating perinatal depression in pregnant refugee and migrant women on the Thai-Myanmar border involved assessing 568 women for depression, using the Structured Clinical Interview for the diagnosis of DSM-IV Disorders (SCID). This study analyses a subsample of 32 women, diagnosed with persistent depression during the antepartum period. Thematic analysis of responses to the SCID and social and demographic surveys was undertaken to investigate factors which contribute towards, or protect against, persistent antepartum depression.

**Results:**

Major themes which women described as contributing towards persistent antepartum depression were financial problems, interpersonal violence, substance misuse among partners, social problems and poor health. Factors women considered as protecting mental wellbeing included social support, accessible healthcare and distractions, highlighting the need for focus on these elements within refugee and migrant settings. Commonly expressed phrases in local Karen and Burmese languages were summarised.

**Conclusions:**

Knowledge of factors affecting mental wellbeing in the study population and how these are phrased, may equip stakeholders to better support women in the study area. This study highlighted the limitations of contextually generic diagnostic tools, and recommends the development of tools better suited to marginalised and non-English speaking groups.

## Background

Depression during pregnancy, also referred to as antepartum depression, affects as many as 15% of pregnant women globally ^1^. Whilst prevalence appears to be higher in women living in low- and middle- income countries (LMICs), available literature is limited in these settings ^2^. Understanding and management of depression in pregnancy is integral to achievement of the third Sustainable Development Goal, which aims towards universal promotion of health and wellbeing, with specific targets to improve maternal and mental health ^3^.

Diagnosis of depression is based on criteria within the Diagnostic and Statistical Manual of Mental Disorders IV (DSM-IV), which include symptoms of low mood, loss of enjoyment, changes in sleep or appetite, slow thought-processing, fatigue, feelings of worthlessness, poor concentration, and suicidal ideation ^4^. The consequences of depression during pregnancy are long-lasting and can affect both mother and child throughout pregnancy, the post-partum period, and beyond. Depressed mothers are more vulnerable to low self-esteem, impeded infant bonding and engagement with poor health behaviour ^5^. Infants of mothers with depression are at a higher risk of being born at a reduced birth-weight, with long-lasting implications for cognitive, emotional and behavioural development ^6^. Furthermore, maternal stress, anxiety and depression have been associated with impaired foetal cerebral growth ^7, 8^, underlining the need for the causes of maternal mental health to be understood, and for any remediation opportunities to be explored.

Factors including stressful life events, absence of social support, domestic violence and dysfunctional partner relationships have been associated with depression during pregnancy ^9^. Pregnant refugee and migrant women are particularly vulnerable to depression due to factors including low socio-economic status, belonging to a minority ethnic group and reduced social support, often compounded by poor access to health services in their place of resettlement ^10^. Previous research into migrant and refugee maternal mental health has largely focused on populations that have relocated to high-income countries (HICs), highlighting a need for further study of populations who have resettled within LMICs ^10, 11^.

Research conducted along the Thailand-Myanmar border by the Shoklo Malaria Research Unit (SMRU) has been among the first to explore depression amongst pregnant refugee and migrant women in a low-income setting ^12^. Fellmeth *et al.* ^13^ conducted a prospective cohort study investigating the prevalence of perinatal depression amongst refugee and migrant women in this region. Participants completed demographic and social surveys, and participated in Structured Clinical Interviews for the Diagnosis of DSM-IV disorders (SCID) during the first, second and third trimesters of pregnancy. These interviews contain rich first-hand accounts of the experiences of depression in the study population, yet have never been qualitatively analysed.

Previous quantitative analysis of this data revealed that the prevalence of moderate-severe perinatal depression in the study population was 18.5% ^13^. Alongside these high rates of depression, there are many barriers to accessing health services along the border. Service provision is minimal on the Myanmar side, whilst women accessing clinics in Thailand often face language barriers, limited means of transportation and a reduced range of services for people without Thai nationality ^14^. To fill this service gap, SMRU provides antenatal clinic (ANC) services along the border. Clinic staff are highly experienced in caring for local migrant and refugee women, and, as members of the communities themselves, are fluent in the major local languages ^12^. In the absence of comprehensive mental health services in this resource scarce setting, a greater understanding of antepartum depression amongst women attending ANC could inform the implementation of future interventions and screening tools, that clinic staff could use to better support the mental health of their patients.

The aim of this study was to provide insight into the perceived contributory and protective factors for persistent antepartum depression in pregnant refugee and migrant women living along the Thai-Myanmar border, in order to better understand their needs and help inform provision of mental health support to this population. We also aimed to explore the timing and frequency with which each depressive symptom was reported, to understand the trajectories of persistent depression in this population, and identify important time-periods for which interventions could be planned. The study also aimed to investigate the ways in which findings were expressed in local languages (Burmese and Sgaw Karen), to increase the usability of study findings for local healthcare workers.

## Methods

This is a secondary analysis of data originally collated by Fellmeth *et al.* ^13^ as part of wider research into the mental health of perinatal migrant and refugee women living along the Thai-Myanmar border. Full details of the methodology undertaken for data collection can be found alongside the primary research ^13^.

The Thai-Myanmar border is home to many refugees and migrants, the majority of whom have fled from long-standing conflict, scarcity of opportunity and instability in Myanmar ^15^. Over 91,000 refugees live along the border ^16^. The largest refugee camp in this area is Mae La camp, Tak Province, Thailand, which houses over 35,000 people ^17, 18^. Mae La is largely populated by people of Karen ethnicity, with smaller populations of Burman and Mon ethnic groups ^17^. The main languages spoken in Mae La are Sgaw Karen, Burmese and Poe Karen ^13^. Tak province is also inhabited by a large and mobile population of migrant workers, often living in remote villages and relocating frequently in search for agricultural and seasonal work ^19, 20^. Although an official labourer recruitment system exists within Thailand, many cross the border informally, fleeing poverty and seeking employment on the Thai side of the border ^21^.

**Figure 1.**
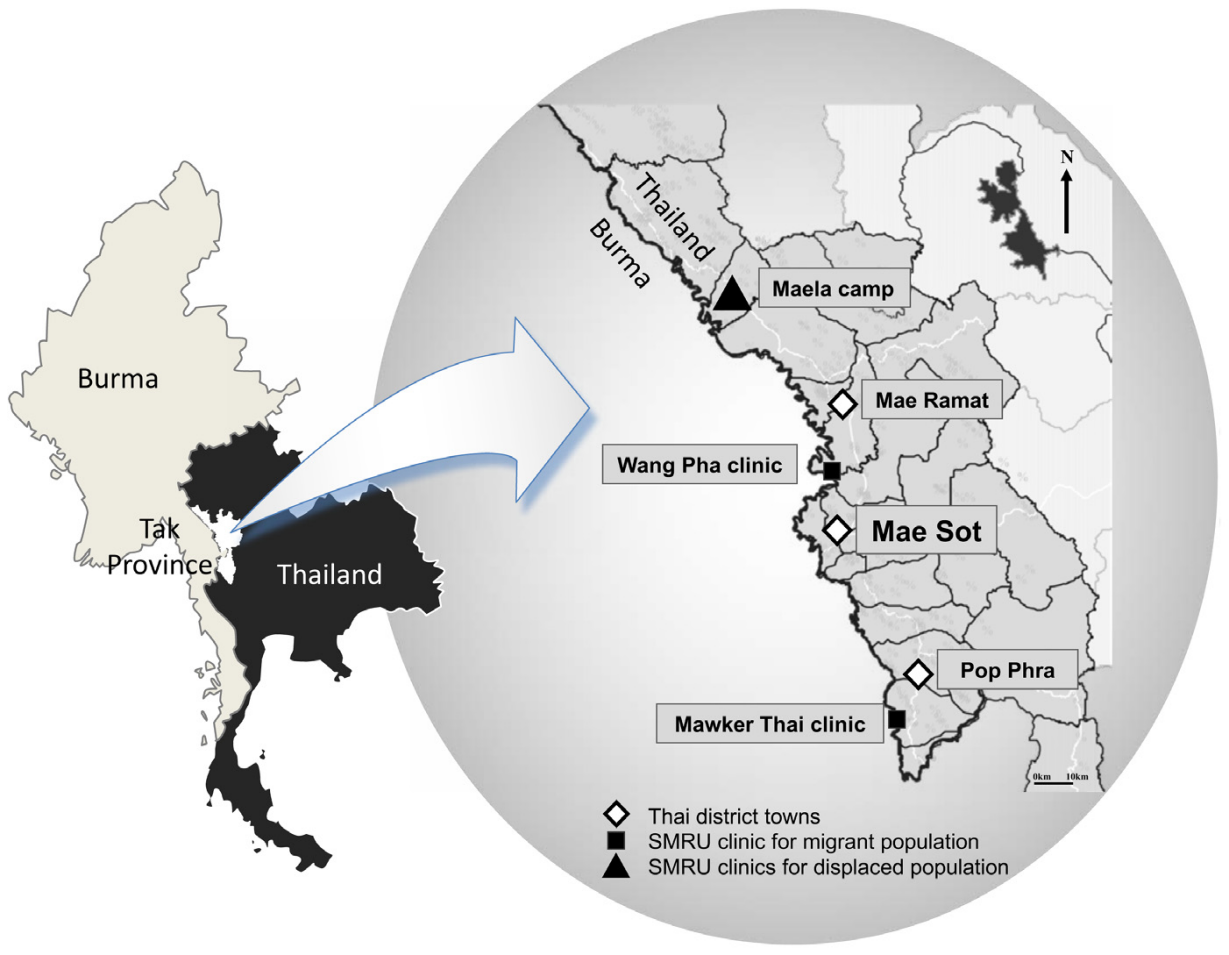
Map of SMRU clinics along the Thai-Myanmar border. This figure has been reproduced with permission from Verena Carrara at SMRU ^19^.

SMRU was established in 1986 as a collaboration between Mahidol University in Thailand, and the University of Oxford in the UK ^13^. Among other activities, SMRU provides reproductive healthcare services including ANC to those living along the Thai-Myanmar border. In December 2016, one clinic was situated in Mae La refugee camp (MLA), and two migrant clinics were located at Wang Pha (WPA) and Mawker Thai (MKT), seen in Figure 1 ^13^.

### Study design

Between October 2015 and April 2016, pregnant women aged 18 or above who attended ANC at MLA, WPA or MKT, and intended to give birth at SMRU, were invited to participate in a cohort study. Women were eligible if their pregnancy was viable and their ultrasound-determined estimated gestational age was below 14 weeks. Out of the 627 eligible women who were approached, 568 women consented to participate in the study.

Participating women were interviewed using the SCID during the first, second and third trimesters of pregnancy, and again during the postpartum period. Interviews were conducted by experienced female healthcare workers (MMO, SN, YP, KW, NK) in either Burmese, Sgaw Karen, Poe Karen, or English, depending on the participant’s preference. Following each SCID interview, women were given a negative or positive diagnosis of depression independently by two physicians, based on DSM-IV criteria ^22^. Positive diagnoses were classified as Major Depressive Disorder, Minor Depressive Disorder, or Depressive Disorder Not Otherwise Specified (NOS). The SCID is traditionally considered a gold-standard tool for diagnosing depression in this population, due to its ease of administration and ability to elicit greater detail compared to alternative instruments ^23^. The SCID assesses the following symptoms: low mood, loss of interest or pleasure, changes in appetite, changes in sleep, restlessness, low energy, feelings of worthlessness, difficulty concentrating, and suicidal thoughts. During the first trimester, an interviewer-completed demographic survey was obtained from each participant. During the third trimester, each participant responded to an interviewer-completed social survey.

The SCID interviews and surveys were carried out verbally in a private room by staff fluent in Sgaw Karen, Burmese, Poe Karen or English. This promoted the participants’ comprehension of the questions, as literacy in the population is low, at 47% ^19^. Subsequently, SCID responses during the first, second and third trimesters were noted in the interview language by the interviewers at the time of interviewing and later translated into English. The responses to the demographic and social surveys were recorded digitally. This culminated in a final electronic dataset, including SCID responses during pregnancy and social and demographic characteristics of the 568 participating women.

### Sampling

For this analysis, a subsample of 32 women was extracted from the original dataset to include all women who were persistently depressed during the antenatal period, defined as women with a positive diagnosis of depression (i.e. either Major Depressive Disorder [severe depression], Minor Depressive Disorder [moderate depression] or Depressive Disorder NOS [mild depression]) at all three trimesters of pregnancy. Women with persistent depression were selected, as these women represented a particularly vulnerable group, likely to have experiences which differed from women with briefer episodes of depression. Women who missed one or more ANC appointment were excluded from the sample. The SCID responses from the postpartum period were excluded; this ensured that the findings reflected the experiences of antepartum depression, as opposed to perinatal depression which may continue up to 12-months post-partum.

### Data analysis

Demographic data was expressed in median (range) and frequency (proportion). The total number of women reporting each symptom of depression was tabulated by trimester of pregnancy. A heatmap to illustrate the severity of depressive symptoms over time for each participant was generated with the R package "ComplexHeatmap" ^24^. All other statistical analyses were done with Microsoft Excel ^25^. SCID transcripts and demographic information were imported into NVivo-12 ^26^ for thematic analysis. Researchers (TAN) conducted thematic analysis in six phases, as described by Braun and Clarke ^27^.

Initially, the SCID transcripts and survey responses were read thoroughly, thus enabling familiarisation with the dataset. Subsequently, researchers assigned codes to specific extracts within the data. Braun and Clarke ^27^ advise researchers to code “interesting features of the data in a systematic fashion” (p.87). The dataset included SCID responses categorised into the nine depressive symptoms defined in the DSM-IV, with a tenth question assessing ability to cope. Responses reporting positive symptoms to questions 1–9 were assigned to a priori codes according to each depressive symptom. Other emergent codes were assigned to factors that women felt contributed to, or protected against, their depressive symptoms.

This study used secondary analysis of SCID interviews and social survey responses to gain an insight into the perceived protective and contributory factors to their experience of persistent antepartum depression. The verbal SCID responses given by women enabled a detailed assessment of which depressive symptoms were commonly experienced in the population, and to which contributory factors these symptoms were frequently connected. The participants’ responses to SCID and social surveys also gave insight into factors that promote their mental wellbeing.

The dataset was re-read once the entirety of the data had been coded, to ensure that extracts had been appropriately coded. For example, some women reporting changes in appetite, sleep and energy, cited non-depressive causes for these symptoms, such as morning sickness, other pregnancy-related symptoms, or temperature during the hot season. These responses were not coded into their corresponding depressive symptom if women attributed them to being pregnant, rather than being related to depression.

Subsequently, themes were derived by grouping coded data into main areas for discussion. The thematic structure was re-visited by researchers several times throughout the analysis process and the write-up of the report. The mapping of themes were discussed regularly, resulting in a final framework that researchers agreed upon. The final themes were defined as: Depressive symptoms, Contributory factors, Protective factors.

## Results

### Participant demographics

**Figure 2.**
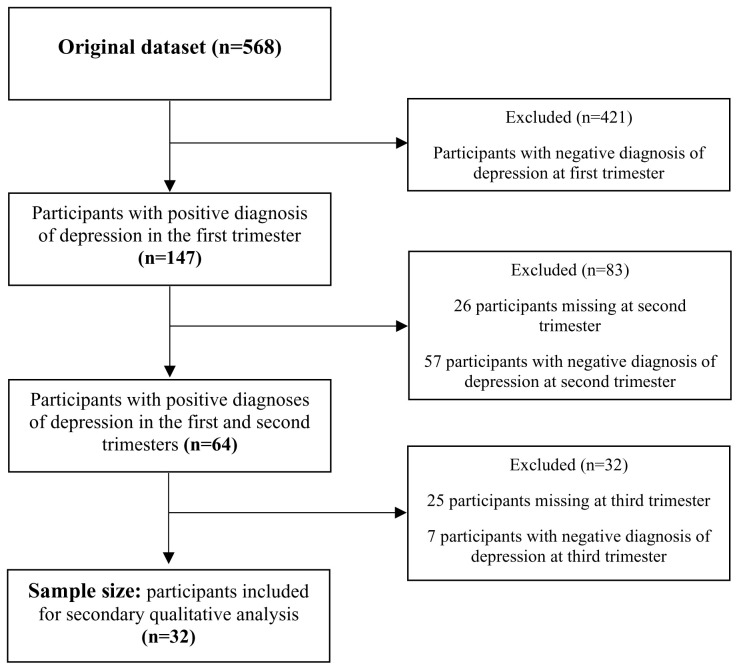
Summary of sampling process.

Figure 2 summarises the sampling process, yielding a sample of 32 women who were persistently depressed throughout pregnancy.

The median age of study participants included in the current analysis was 26 years, with a range of 18 to 39 years. Over half (n=18, 56.3%) of the participants were refugees, accessing SMRU antenatal care at MLA. The remaining participants accessed antenatal care at SMRU migrant clinics: nine at (28.1%) WPA and five at (15.6%) MKT. Among the refugee population, the majority of study participants were of Sgaw Karen ethnicity (n=12). Among the labour migrant community, the most common ethnicity was Burman (n=7). Buddhism was the most common religion in both refugee (n=7) and migrant (n=12) populations. Over half (n= 18) of all women classed their occupation as ‘Home’. A majority of women were married, with just one participant ‘cohabiting’. Eighteen (56.3%) women replied ‘Yes’ when asked ‘Have you ever suffered from mental illness?’ in the demographic survey.

### Prevalence of depressive symptoms

**Figure 3.**
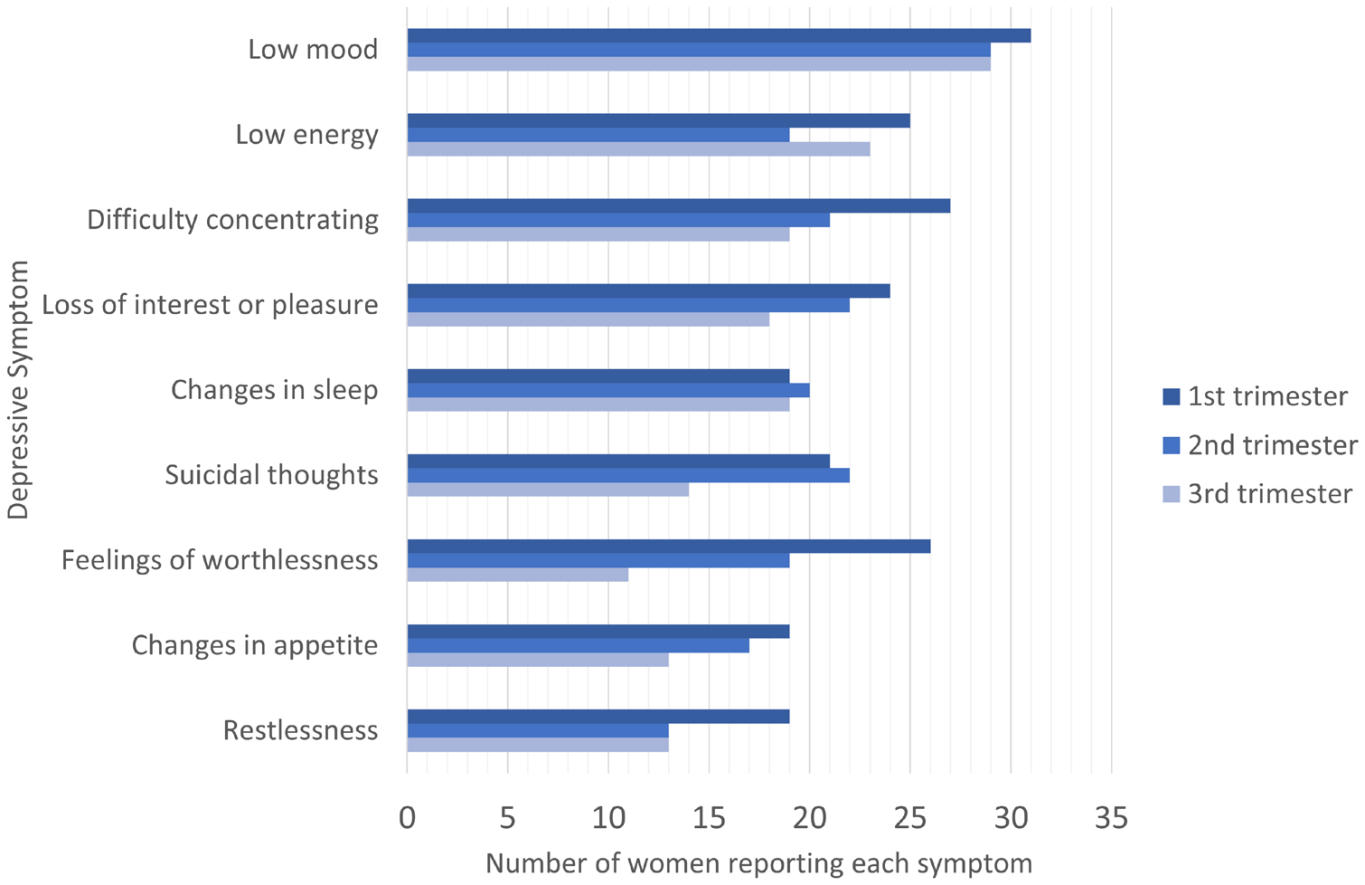
Number of women reporting each symptom during the three trimesters of pregnancy.

The most commonly reported depressive symptom was low mood, with 96.9% (31 of 32), 90.6% (29 of 32) and 90.6% (29 of 32) women reporting this symptom in the first, second and third trimesters, respectively. Other common symptoms were low energy (n=25, 19, 23) and difficulty concentrating (n=22, 24, 18). Figure 3 summarises the number of women reporting each symptom across the three trimesters.

In general, the number of women reporting each depressive symptom declined throughout pregnancy. Figure 3 shows that symptoms including difficulty concentrating, suicidality, changes in appetite, and restlessness, were reported less frequently as the pregnancy progressed. Other symptoms including low mood and the feeling of worthlessness, persisted throughout.

### Severity of depression

**Figure 4.**
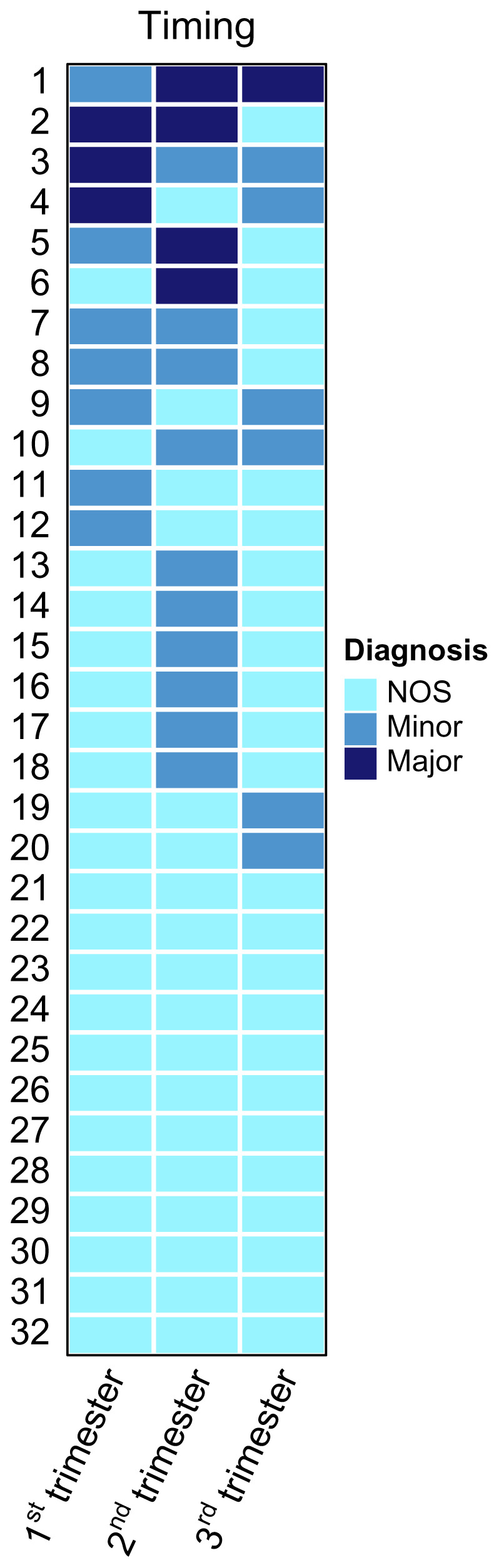
Heatmap displaying the depression diagnosis each woman in the study sample (n=32) received at each trimester.

The heatmap in Figure 4 shows severity of women’s depression over time. The majority of women experienced either persistent mild depression (n=12) or a fluctuation between mild and moderate depression (n=14). Only a small proportion of women experienced severe depression at one or more timepoints (n=6). The number of women with severe, moderate and mild depression in the first trimester was 3, 7 and 22, respectively. The number of diagnoses given for severe and moderate depression increased in the second trimester (n=4, 10, 18), but decreased in the third (n=1, 6, 25).

### Contributory factors

**Figure 5.**
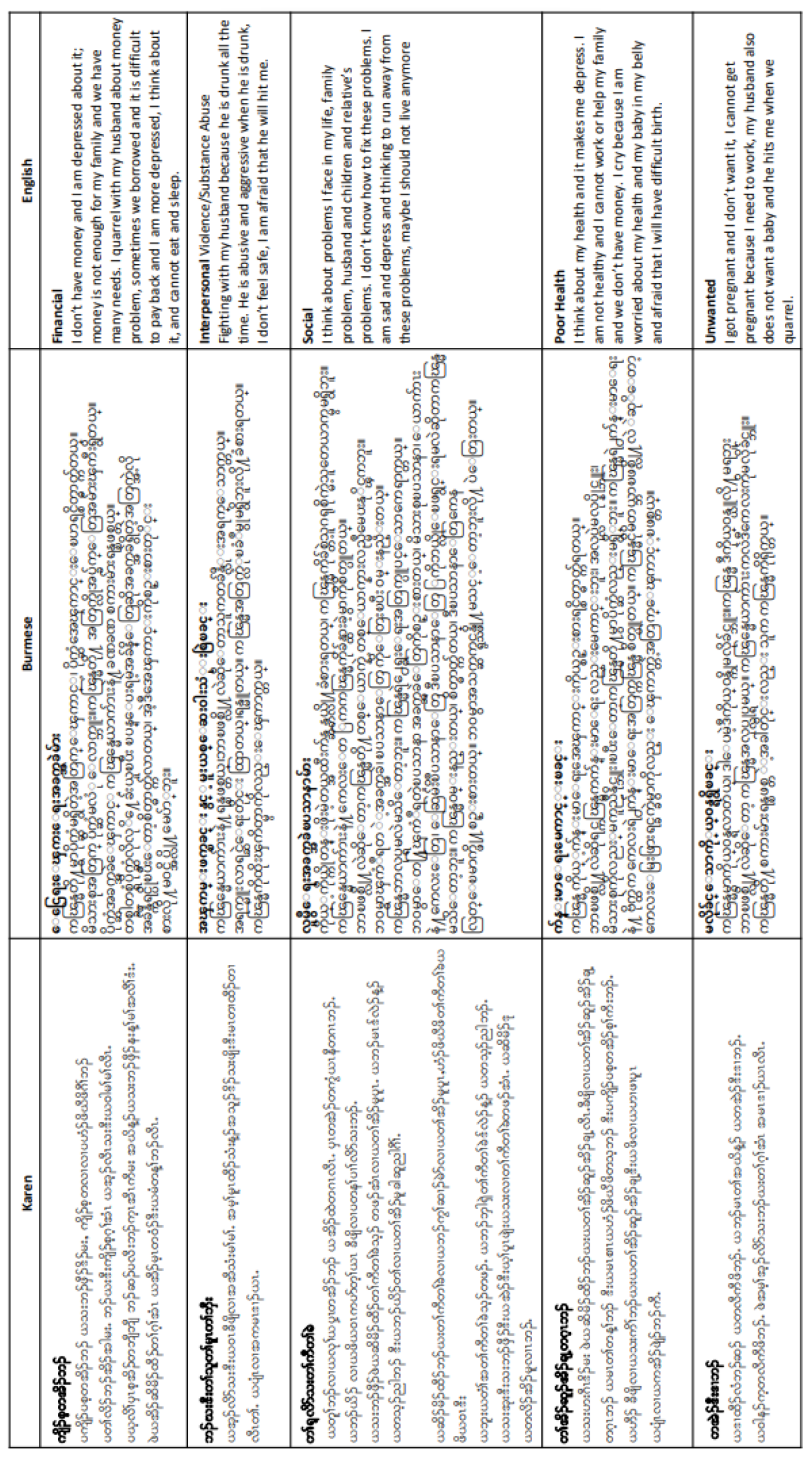
Summaries of common phrases used to describe depressive symptoms in Sgaw Karen, Burmese and English.

Qualitative analysis of women’s interviews revealed that financial problems, interpersonal violence and substance misuse among partners, social problems and poor health were key themes that women felt were contributing to antepartum depression. The phrases used by these women have been summarised in Sgaw Karen and Burmese by local health workers (See Figure 5).

**Financial problems** The most frequently described factor which women felt contributed to their depressive symptoms was financial concerns. Financial problems were most commonly linked to feelings of worthlessness, with many women stating that lack of money made them feel sad, unwanted or incapable:

"Sometimes when my children are asking for snacks or an umbrella and I cannot buy it for them, I feel sad." (Participant 477, age 31, parity 3, MLA refugee clinic)
  "[I feel worthless] because my father came to visit but my brother did not tell me or contact me, so I thought that because I don’t have money they didn’t want to see me." (Participant 24, age 34, parity 5, MKT migrant clinic)

Many women associated financial worries with poor sleep. Commonly, women described lying awake, worrying about whether they would have enough money to care for their family. Several women were specifically concerned about how they would manage after the birth of their child:

"Sometimes [I have a low mood] because my children have to go to school but [I have] no money, thinking I cannot take care of my new baby because no money [sic]." (Participant 373, age 21, parity 1, MLA refugee clinic)

Several women linked their experiences of antepartum depression with concerns about debt, or partners being unable to provide for them:

"Sometimes I feel sad. . . if I want to eat, and my husband cannot take care of me."
  "I thought [about suicide] once, when people asked for my husband[‘s] debt." (Participant 173, age 18, parity 0, WPA migrant clinic)

In some cases, the underlying reason for a woman’s financial concerns was related to their refugee or migrant status:

"I want to find work outside [the camp], but to leave the camp, I have to pay 200 Baht for a permit, then I have no more money to use outside. I want to find money but [it is] difficult." (Participant 333, age 21, parity 0, MLA refugee clinic)

**Violence and substance misuse** Many women were victims of intimate partner violence, and explained how violent episodes contributed to depressive symptoms.

"I’m mostly feeling depressed because of [my] husband. I got abused by my husband. Rib broken." (Participant 248, age 39, parity 1, WPA migrant clinic)

In most cases, interpersonal violence was linked to substance misuse. Very few women reported personal alcohol consumption (n=2), but many women reported incidences where their husbands would drink alcohol, leading to conflict and sexual violence:

"My husband hits me a lot: 8-10 times per month. When he drinks alcohol, but also without alcohol he hits me." (Participant 549, age 20, parity 1, MLA refugee clinic)
  "[I feel sad] because [we have] not enough money and my husband drinks alcohol, after drinking he comes back to fight with me and hit me." (Participant 549)
  "Sometimes I have difficulty breathing. I feel things but cannot say it to anyone. . . My stepfather drinks a lot of alcohol. He beat me and my mother in the past. Once he tried to rape me." (Participant 341, age 24, parity 1, MLA refugee clinic)

Other women reported how substance misuse by their partner or family member can trigger depressive symptoms in the absence of violence.

"I was thinking about suicide because my husband drinks a lot and didn’t care about me and the family." (Participant 113, age 31, parity 3, MKT migrant clinic)

In a few cases, the underlying reason for violence was due to the woman’s pregnancy being unwanted by their partner:

"My husband hit me when we argued - because he didn’t want this baby." (Participant 274, age 23, parity 2, WPA migrant clinic)

Conflict was not limited to intimate partners. Violence between family and friends was commonly described to contribute to suicidal thoughts, or attempts:

"I took medicine for suicide after my mother was angry with me and spanked me." (Participant 85, age 26, parity 1, MKT migrant clinic)

**Social problems** The range of social problems affecting women was considerably diverse, spanning from difficult family relationships, feelings of rejection from family, and concern for loved ones. There were several women who explained that inter-family problems were significant enough to contribute to suicidal thoughts:

"I think about [suicide] sometimes. [In] my side of the family: problems. But [in] my husband’s side: also problems. So I feel in the middle. I think maybe suicide will mean no more problems." (Participant 93, age 24, parity 2, MKT migrant clinic)
  "My parents divorced, my mother got a new husband. Then I felt unhappy, sad, depressed, [I attempted] suicide by drinking a chemical for killing insects." (Participant 24)

Other women linked their feelings of low mood and worthlessness to their experience of feeling neglected by family members:

"Yes, I feel worthless often. My parents, parents-in-law, siblings, husband - nobody loves me so I feel worthless." (Participant 549)
  "Sometimes I want to die. I take care of all my family, but nobody takes care of me." (Participant 249, age 28, parity 0, WPA migrant clinic)

Concern for loved ones stemmed from two main underlying factors: intimate partner infidelity and family members who live far away due to factors related to migration.

"I worry for my husband. He went to stay with his first wife for nearly 15 days and hasn’t come back." (Participant 274)
  "Sometimes I can’t sleep, I lie awake all night, worried about family. . . My mother and father are in Burma, sick." (Participant 320, age 28, parity 2, MLA refugee clinic)

**Poor health** Health conditions, affecting both themselves and their families, were a commonly reported source of depressive symptoms in study participants.

"My husband’s health is not good. Mother ill. When thinking more, I want to cry." (Participant 373)
  "I feel worried for my mother and father-in-law, about how they will live because they cannot see anymore." (Participant 248)

One woman, living with a chronic condition, attributed her suicidal thoughts and restlessness to poor health:

"When I feel sick I want to die. Only thinking about it." (Participant 488, age 23, parity 1, MLA refugee clinic)
  Sometimes I can’t sit still. Something inside me makes me worry, health problems. (Participant 2, age 24, parity 2, MKT migrant clinic)

### Protective factors

**Figure 6.**
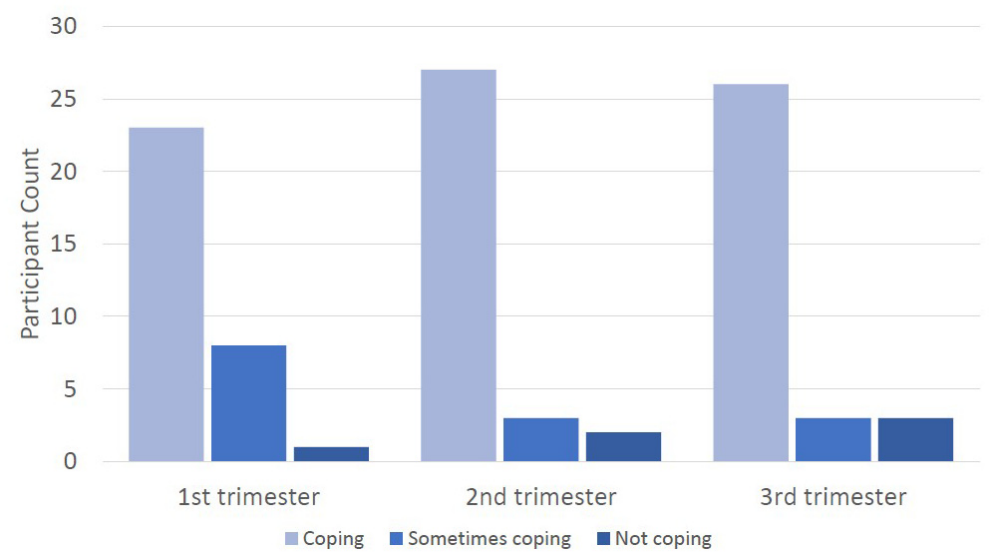
Graph showing the count of participants expressing ability to cope with normal work and home activities.

Question 10 of the SCID asked women about their ability to cope with work and household activities. Although expressed ability to cope was lowest in the first trimester, most women reported that they were able to cope throughout the pregnancy (see Figure 6).

Many women disclosed protective factors against depressive symptoms; social support, healthcare and distraction emerged as main themes.

**Social support** Many women reported that they still enjoy spending time with others, and seek comfort from friends and family:

"I don’t want to do anything but I still enjoy visiting friends." (Participant 173)
  "I feel a little bit better today because I spoke to my sister." (Participant 24)

Whilst the majority of women reported their husbands’ actions as damaging or upsetting, a few women received support and encouragement from their husbands:

"I have no friends to talk to when I am sad but I can talk to my husband, he supports [me]." (Participant 453, age 23, parity 2, MLA refugee clinic)

Fear for the safety of the children of refugee and migrant women was the most common protective factor against suicide:

"Sometimes [I think about suicide], but then think what will happen to my son. I think insecticide can be an easy death, but then husband is alcoholic and will not look after our son." (Participant 341)

Social support was the second most common protective factor for women expressing suicidal thoughts, with several attempts being stopped by friends or family:

"I think about suicide when I fight with my husband. I tried to. . . but my friend stopped me." (Participant 533, age 30, parity 1, MLA refugee clinic)

**Healthcare** Staff and medical care were cited as protective factors against poor mental health. Some women reporting an improvement in mental health connected this with support from SMRU staff:

"Now, since talking to staff at SMRU, I don’t think about suicide anymore." (Participant 261, age 33, parity 0, WPA migrant clinic)
  "I feel I can release the tightness in my chest by telling staff who are listening so patiently." (Participant 370, age 26, parity 4, MLA refugee clinic)

Others expressed an improvement of energy levels or mood after starting clinical treatment.

"I feel better generally. . . I am happier since taking medication." (Participant 477)

In one case, a woman who had been a victim of sexual abuse explained how the presence of local non-governmental organisations (NGOs) serves as a deterrent for further abuse, because the perpetrator “daren’t do anything” in the camp.

**Distraction** Other women explained that they found it easier to cope when they were busy with other activities. In particular, cooking, shopping, washing and working distracts women from thinking about their problems.

## Discussion

The number of depressive symptoms reported by each woman was highest in the first trimester, and declined throughout pregnancy. Additionally, the number of women diagnosed with more severe depression was highest in the second trimester, and lowest in the third. Although the small sample of 32 participants limits the generalisability of findings, this may indicate that women are more vulnerable to depressive symptoms earlier in pregnancy, and thus may benefit from additional support during this time. A study by Behera and Sikary ^28^ found that suicide in pregnant women was most common in the first and second trimesters, indicating that mental health support may be most valuable at this time. Fellmeth *et al.* found prevalence of depression to decline throughout pregnancy in this population ^22^. Research by Choi *et al.* ^29^ found that the majority of women studied experienced a consistent level of depression throughout the perinatal and postnatal period: only 7% of 899 women experienced a decline in depression across pregnancy. This discrepancy may be related to the study location, within an HIC. Contextuality of mental health problems may differ by population; limiting comparability of trends in HICs and LMICs, and futhermore with marginalized refugee and migrant women. Further research into the prevalence of depression over the course of pregnancy in LMICs is merited to inform the time period during which support provision should be focused.

Refugee and migrant women with persistent depression most commonly reported experiencing low mood. As low mood is the mainstay depressive symptom in the Global North, this finding suggests comparability in experiences of depression between this population and higher-income settings ^30^. However, qualitative analysis of how the study population experiences depression is limited due to the SCID responses being framed around the nine DSM-IV symptoms. Depressive symptom profiles vary widely across different cultures; the SCID, a diagnostic tool developed in the Global North, may fail to capture all depressive symptoms that reflect the ‘cultural concept of distress’ in this population ^30, 31^. The translation of SCID questions into Sgaw Karen, Poe Karen and Burmese may have further limited the tool’s ability to capture depression in the population; when local staff summarised common phrases used by refugee and migrant women, the length and complexity required to convey the same information varies widely between each language (see Figure 5). Nevertheless, capturing wording and phrasing used by the women is an important finding for stakeholders working locally on maternal depression. The summary has been used in workshops for local NGO and Community Based Organisation (CBO) staff, such as at the MPHSS (Mental Health and Psychosocial Support) course, conducted by Sayar Nyi Wynn Soe in Burmese. These courses were particularly essential during the influx of displaced persons from Myanmar in the first 6 months of 2022. The empowerment of local communities through communication of study findings is a significant and crucial outcome for this study ^32^.

Financial problems were frequently associated with depressive symptoms in the study population. A systematic review by Fisher *et al.* ^2^, focusing on perinatal mental health in LMICs, found that lower socio-economic status was associated with a higher risk of poor perinatal mental health, supporting the suggestion that women experiencing financial hardship may be more vulnerable to antepartum depression. Although the specific drivers for financial hardship along the Thai-Myanmar border were not thoroughly explored, problems likely emerge in-part due to the Thai government resisting ‘local integration’ of refugees and migrants, leading to difficulty obtaining regular, stable employment ^33^. Furthermore, less than 50% of women had paid employment during pregnancy. This phenomenon is in part due to the employment of refugees in the host country being officially illegal, child care services relying on family members instead of formal services for migrant women, and women’s commitment to, and traditional role within, child care. Nevertheless, barriers to women contributing financially to the family may increase the likelihood of monetary insecurity and feelings of worthlessness that become heightened with pregnancy. Solving the wider issue of economic and political instability in the area is an aim towards which NGOs and other stakeholders in the area are currently working ^34^. However, increased awareness of the impact of these issues, and increased advocacy amongst staff, will serve to support the study population.

Analysis of SCID data also revealed that interpersonal violence and substance misuse among women’s partners were common factors contributing to depressive symptoms in women, with suicidal thoughts being of particular concern. These factors should be viewed as key target areas for mental health support. A qualitative study by Fellmeth *et al.* ^35^, conducted in the same setting as for the original dataset, similarly found that alcohol use in partners was common and led to feelings of shame, stress, and violence. *Falb et al.* ^36^ found that 9.3% of refugee women living along the Thai-Myanmar border had been victim to one or more forms of violence. Fisher *et al.* ^2^ similarly found that alcohol use and violence within intimate partner relationships were linked to a higher prevalence of poor mental health during pregnancy. It is evident that violence and alcohol misuse in lower-income settings is an important issue for organisations to be aware of, and to act upon. The UNHCR addresses gender-based violence (GBV) within refugee camps, recommending remediation through community involvement, male partner engagement, and implementation of staff training ^37^. However, such programmes were not available for migrant women in this cohort and may not be universally appropriate to implement; Horn found that community action against GBV was often rejected by inhabitants of Kakuma refugee camp, Kenya ^38^. This highlights the importance of assessing contextual compatibility of recommendations. Further research into which activities would be most effectively implemented along the Thai-Myanmar border would be essential to improving mental health support to pregnant refugee and migrant women in the area.

Protective factors which emerged from analysis highlight important elements to promote within the study population. Social support, healthcare and distractions all served to improve the mental wellbeing of pregnant women. Research in both HICs and LMICs indicate the benefits of social support to good antepartum mental health ^12, 39, 40^. Further research into how social networks can be promoted within refugee communities would benefit the study population. Several women highlighted the positive impact of the support that they had received from healthcare staff. Raising staff awareness of the value of listening to pregnant migrant and refugee women with antepartum depression may lead to this support becoming more available to women accessing ANC.

### Strengths and limitations

In-depth analysis of SCID data provided a detailed understanding and insight into experiences of depression in this population. The use of the SCID, which uses open questions and allows for nuanced discussions, enables more information to be conveyed by those experiencing depression, and provides a more sensitive measure than other means, including standardised screening tools. Nevertheless, even the SCID is structured around a set of depression symptoms defined in high-income settings within the Global North; therefore, there may remain a number of trans-cultural deficiencies relating to the ways in which depression was ascertained in this study. Three-language availability of the major ways women may express their problems remains an important outcome for raising local awareness.

Women who missed one or more ANC appointment were excluded from the subsample. This may have skewed the sample to include more women who were permanently settled in the study location, and therefore more able to attend all ANC appointments. Severe symptoms may have restricted a woman’s ability to cope with attending ANC, resulting in a missed appointment. This may have led to the exclusion of participants with insightful experiences of antepartum depression, although this was a necessary concession to examine trends.

Furthermore, in Fellmeth’s study, participants responding to the SCID may have been ‘primed’ during the second and third trimesters, resulting in respondent bias in which responses are influenced by previous SCID interviews. SCID interviews may be considered to be of therapeutic value in themselves, by providing women with an opportunity to talk about their mental health ^13^.

Changes in appetite, sleep pattern or energy levels were not coded under ‘Depressive symptoms’ if they were described by participants as being unrelated to pregnancy. However, occasionally the underlying reason for these symptoms was unclear, which may have resulted in falsely high or low counts of these symptoms. Additionally, a few responses in the dataset were cut short, with missing letters. Although this was uncommon, some valuable information may be missing. Due to the dataset being anonymised and retrospective, clarification of meaning through respondent validation was impossible, reducing the validity of analysis ^41^.

In Karen culture, it is uncommon to freely discuss personal challenges and burdens to people outside of close family. Such disclosure is often avoided in order to prevent concern and gossip within the community. Due to this social norm, it was unusual for some women to openly share their thoughts during their interview, which may have led to respondent bias. This effect was mitigated by ensuring that interviews were conducted by highly trained staff who were known to, and culturally integrated within, the study population, increasing the potential for a trusting rapport between the women and their interviewer ^13^.

## Conclusions

This study provides insight into the experiences of persistent antepartum depression amongst pregnant refugee and migrant women living along the Thai-Myanmar border and highlights the need for awareness of these issues amongst health care staff working in the area. Specifically, staff should be aware of the substantial barriers to good mental health facing these women, including financial concerns, violence, substance misuse, social problems and poor health. Furthermore, initiatives that promote protective factors including social support, healthcare provision and distraction should be encouraged and invested in to help support pregnant women’s mental wellbeing. The trajectory of antenatal depression in this population suggests that severity of depression may decline at the end of pregnancy. Further research into the underlying factors affecting the course of depression throughout pregnancy may inform when, and in what way, support can be most effectively offered. Future use of heatmap visualisation could be used to guide the timing of support provision. Increased multi-sectoral advocacy to tackle the issues of unemployment, financial scarcity and GBV along the Thai-Myanmar border is merited. Development of a context-specific and locally-validated screening tool to identify pregnant refugee and migrant women at risk of antepartum depression may help to identify women in need of support. This tool should be developed in the native language of local population and reflect culturally-relevant experiences of depression.

## Ethics statement

Ethical approval for the original prospective cohort study was granted by the Oxford Tropical Research Ethics Committee (OxTREC 28-15), the Tak Border Community Advisory Board (T-CAB 6/2/2015) and the Mahidol University Faculty of Tropical Medicine Ethics Committee (TMEC 15-045) ^13^. Ethical approval for secondary analysis of the dataset was granted by the Leeds Institute of Health Sciences Research Ethics Sub-Committee (FMHREC-20-2.2).

## Consent

Written informed consent for publication of participant responses was obtained from the participants ^13^.

## Data Availability

The data associated with the original study by Fellmeth *et al.* ^13^, is not publicly available. Proposals for collaboration are welcomed, and detailed enquiries should be directed to the corresponding author. Open Science Framework: COREQ checklist for: ’Persistent depression in pregnant refugee and migrant women living along the Thai-Myanmar Border: a secondary qualitative analysis.’, https://doi.org/10.17605/OSF.IO/UWXQA ^42^. Data are available under the terms of the Creative Commons Zero "No rights reserved" data waiver (CC0 1.0 Public domain dedication).
