# Supplementary material for: Persistent depression in pregnant refugee and migrant women living along the Thai-Myanmar Border: a secondary qualitative analysis
Source: Wellcome Open Res. 2024 Mar 28;7:231. Originally published 2022 Sep 12. [Version 2] doi: 10.12688/wellcomeopenres.17744.2 (PMC11459118; doi:10.12688/wellcomeopenres.17744.2)
Supplement: Peer review history

# Reviewer report: Reviewer response for version 2

**DOI:** 10.21956/wellcomeopenres.23429.r95679

**Published:** 2024-10-07

**Authors:** Surendran Rajaratnam

**Affiliations:** 1 Faculty of Social Sciences and Humanities, Universiti Kebangsaan Malaysia, Bangi, Selangor, Malaysia

Thank you for the opportunity to review the manuscript. It was a pleasure to contribute to the review process, and I appreciate being involved in evaluating such an important study. I hope my comments will help strengthen the article and contribute to its successful indexing. Below are some of the areas of improvement that can be considered by the authors of the paper. 

- **Demographic Table for Participants:**
  The manuscript would benefit from including a table summarizing the demographic characteristics of the participants. This information is crucial as it helps readers better understand the participants composition and any potential factors that could influence the results.
- **Discussion Section Lacks Engagement with Recent Studies:**
  The discussion section requires further engagement with recent literature. While the findings are valuable, they are not sufficiently framed in the context of recent studies, which limits the scope of the discussion. By comparing your results with up-to-date research, you would better identify gaps in the current knowledge and potential directions for future research.
- **Findings Not Discussed in Relation to Pregnancy Trimesters:**
  There is a lack of linkage between the themes from the study’s findings and the three trimesters of pregnancy. Since in the article emphasizes on this aspect of pregnancy and its relation to depressive symptoms, relating the findings to the specific experiences of participants during these periods would provide a deeper and more structured understanding of the data. It is suggested that the authors revise the discussion to explicitly connect the themes of your study to each trimester as much as possible (if the data that was collected allows for this level of analysis) for a more nuanced interpretation of your results.
- **Separate Discussion and Limitations Sections:**
  The manuscript currently combines the discussion of the findings with some of the study’s limitations. These two aspects should be clearly delineated. The discussion section should focus solely on interpreting and analysing the findings in light of past studies, while the limitations section should focus on the constraints or challenges in conducting and analysing the data for the study. Clearly distinguishing these sections will improve the overall structure and coherence of the manuscript, allowing readers to understand the findings without being immediately confronted by limitations.

Is the work clearly and accurately presented and does it cite the current literature?

Partly

If applicable, is the statistical analysis and its interpretation appropriate?

Not applicable

Are all the source data underlying the results available to ensure full reproducibility?

No

Is the study design appropriate and is the work technically sound?

Yes

Are the conclusions drawn adequately supported by the results?

Yes

Are sufficient details of methods and analysis provided to allow replication by others?

Yes

Reviewer Expertise:

Gender inequalities and women's empowerment, social work in rural communities, healthcare as well as with refugee and migrant communities.

I confirm that I have read this submission and believe that I have an appropriate level of expertise to confirm that it is of an acceptable scientific standard.

# Reviewer report: Reviewer response for version 1

**DOI:** 10.21956/wellcomeopenres.19637.r57603

**Published:** 2023-11-27

**Authors:** Magdalena Chrzan-Dętkoś

**Affiliations:** 1 University of Gdańsk, Gdańsk, Poland

Thank you for the possibility of reviewing this interesting article. Overall, there are 32 countries currently in conflict.  While the severity and duration of these conflicts differ, they all have significant impacts on the affected populations, with women in the perinatal period being in additionally vulnerable situations. The work of the authors, apart from the scientific significance, can bring awareness of these conflicts and the situation at the Thai - Myanmar Border. The aims to minimize the likelihood of re-traumatization of refugee pregnant women and new mothers are of the highest priority and we can believe that describing the situation via studies can help policymakers to support these women. Mitigating stress and promoting resilience through providing trauma-informed and migration-informed perinatal mental health care is crucial for the neurobiological and emotional-behavioral mother-baby interactions. The authors emphasize cultural differences and the need to respect them - which is also very important. Thank you for your important work.

Is the work clearly and accurately presented and does it cite the current literature?

Yes

If applicable, is the statistical analysis and its interpretation appropriate?

Yes

Are all the source data underlying the results available to ensure full reproducibility?

Partly

Is the study design appropriate and is the work technically sound?

Yes

Are the conclusions drawn adequately supported by the results?

Yes

Are sufficient details of methods and analysis provided to allow replication by others?

Yes

Reviewer Expertise:

Psychology, perinatal mental health

I confirm that I have read this submission and believe that I have an appropriate level of expertise to confirm that it is of an acceptable scientific standard.

# Response

**Published:** 2024-02-29

**Authors:** Tabitha Ashley-Norman

**Affiliations:** University of Leeds, UK

Thank you so much for your peer review and appraisal of our paper. We are very grateful for your comments summarising the importance of this research.

# Reviewer report: Reviewer response for version 1

**DOI:** 10.21956/wellcomeopenres.19637.r57602

**Published:** 2023-07-04

**Authors:** Bronwyn Myers

**Affiliations:** 1 Curtin EnAble Institute, Curtin University, Perth, Western Australia, Australia

Thank you for the opportunity to comment on this well-written and interesting manuscript. The paper examines commonly reported symptoms of depression among pregnant refugee and migrant women and their perceptions of factors that contribute to and diminish these symptoms of depression. I have several suggestions for strengthening the manuscript that I hope the authors will find useful.  

 First, in the background, it would be useful to describe how prepartum depression can persist into the post-partum period and how this may impact on mother-infant bonding, infant growth and development etc. 

 Second in the background, please explain how a greater understanding of commonly experienced symptoms of depression in this study will equip health workers with knowledge on how to work more effectively with this vulnerable group of pregnant women. Will this information be used to inform future health worker training programs or other intervention efforts?

 Third (background), please provide some context and a rationale for the second and third aims of this study. There was a clear rationale for the first aim. I was not clear on why the second and third aims were necessary or how these related to the overarching goal of the project.

 Fourth, for the methods, it would be helpful to describe the SCID and the open-ended questions/responses here rather than in the discussion.

 Fifth (Results). There are a lot of tables and figures- some of which repeat results described in the text. I think Table 1 can be removed. Consider removing some of the figures or combining  to reduce the number.  It may be possible to remove figure 5 without losing any key information from the paper.

 Sixth, For the excerpts from the transcripts- please can you attribute each quote to a specific participant and add some descriptors (age, parity, migrant or refugee status) 

 Finally, you have noted that the SCID is generic and as it was designed in the global north may not capture cultural explanations of prenatal depression and as such your study may have missed these idioms and explanations. What do you think the SCID missed? Are there questions or areas where the SCID could be expanded to capture these cultural explanations?

Is the work clearly and accurately presented and does it cite the current literature?

Yes

If applicable, is the statistical analysis and its interpretation appropriate?

Not applicable

Are all the source data underlying the results available to ensure full reproducibility?

No source data required

Is the study design appropriate and is the work technically sound?

Yes

Are the conclusions drawn adequately supported by the results?

Partly

Are sufficient details of methods and analysis provided to allow replication by others?

Partly

Reviewer Expertise:

mental health interventions, vulnerable populations, access to mental health interventions

I confirm that I have read this submission and believe that I have an appropriate level of expertise to confirm that it is of an acceptable scientific standard, however I have significant reservations, as outlined above.

# Response

**Published:** 2024-02-29

**Authors:** Tabitha Ashley-Norman

**Affiliations:** University of Leeds, UK

Thank you for your thoughtful and detailed comments on our manuscript. We are grateful for your guidance and have made some relevant changes to our new version, which we have outlined for you below. We have included your original suggestions for clarity. Suggestion 1:

 First, in the background, it would be useful to describe how prepartum depression can persist into the post-partum period and how this may impact on mother-infant bonding, infant growth and development etc.  Response 1: Thank you for this suggestion. The following paragraph within the Background section now more explicitly links pre-partum depression to long-lasting impacts: *The consequences of depression during pregnancy are long-lasting and can affect both mother and child throughout pregnancy, the post-partum period, and beyond. Depression during pregnancy can persist post-partum, and mothers with depression are more vulnerable to low self-esteem, impeded infant bonding and engagement with poor health behaviour 5 . Infants of mothers with depression are at a higher risk of being born at a reduced birth-weight, with long-lasting implications for cognitive, emotional and behavioural development 6 .*

   Suggestion 2:

 Second in the background, please explain how a greater understanding of commonly experienced symptoms of depression in this study will equip health workers with knowledge on how to work more effectively with this vulnerable group of pregnant women. Will this information be used to inform future health worker training programs or other intervention efforts? Response 2: We have added clarity to the potential utilisation of our findings in the below paragraph (within the Background section): *In the absence of comprehensive mental health services in this resource scarce setting, a greater understanding of women’s experiences of depression could inform the development of screening and care pathways by highlighting key stressors which could be targeted. A better understanding of women’s needs and experiences will enable clinic to better support the mental health of their patients.* Suggestion 3:

 Third (background), please provide some context and a rationale for the second and third aims of this study. There was a clear rationale for the first aim. I was not clear on why the second and third aims were necessary or how these related to the overarching goal of the project. Response 3: The description of aims now more clearly explains the rationale for the second and third aims below (in the Background section): *We also aimed to explore the timing and frequency with which each depressive symptom was reported, to understand the trajectories of persistent depression in this population, and identify important time-periods for which interventions could be planned. The study also aimed to investigate the ways in which findings were expressed in local languages (Burmese and Sgaw Karen), to increase the usability of study findings for local healthcare workers.*

   Suggestion 4:

 Fourth, for the methods, it would be helpful to describe the SCID and the open-ended questions/responses here rather than in the discussion. Response 4: A description of the SCID responses has been moved from the discussion to the methodology now, within the ‘data analysis’ section, as suggested.

   Suggestion 5:

 Fifth (Results). There are a lot of tables and figures- some of which repeat results described in the text. I think Table 1 can be removed. Consider removing some of the figures or combining  to reduce the number.  It may be possible to remove figure 5 without losing any key information from the paper. Response 5: Thank you for our suggestion. We have removed Table 1 and instead listed the depressive symptoms assessed for in the SCID in the text, for readers less familiar with the DSM-IV criteria (see Study Design, paragraph 2). Figure 5 we feel is important in our setting (and in global locations where Burmese and Karen have been resettled – note resettlement programs from the Thailand Myanmar Border have been one of the largest such UNHCR projects) as it is helpful for the local health workers to see the phrases used in each of the local languages verbatim. We therefore feel it is an important component of the manuscript and would like to retain it.

   Suggestion 6:

 Sixth, For the excerpts from the transcripts- please can you attribute each quote to a specific participant and add some descriptors (age, parity, migrant or refugee status)  Response 6: This has now been done Suggestion 7:

 Finally, you have noted that the SCID is generic and as it was designed in the global north may not capture cultural explanations of prenatal depression and as such your study may have missed these idioms and explanations. What do you think the SCID missed? Are there questions or areas where the SCID could be expanded to capture these cultural explanations? Response 7: The SCID is generic and designed in the global north and is therefore may be limited for detection of depression. Certain cultural idioms remain difficult to map onto the SCID criteria including symptoms of ‘heavy heart’ and ‘thinking too much’ and vague physical manifestations – ‘heavy neck’, tingling and head pain (not headache). Future studies open to recognising cultural idioms will improve SCID as a global tool.   Thank you again for your suggestions.
